# Analyses of the expression, immunohistochemical properties and serodiagnostic potential of *Schistosoma japonicum* peroxiredoxin-4

**DOI:** 10.1186/s13071-020-04313-w

**Published:** 2020-09-01

**Authors:** Minh-Anh Dang-Trinh, Jose Ma. M. Angeles, Kharleezelle J. Moendeg, Adrian Miki C. Macalanda, Thu-Thuy Nguyen, Luna Higuchi, Shotaro Nakagun, Masashi Kirinoki, Yuichi Chigusa, Yasuyuki Goto, Shin-ichiro Kawazu

**Affiliations:** 1 National Research Center for Protozoan Diseases, Obihiro University of Agriculture and Veterinary Medicine, Hokkaido, Japan; 2 The United Graduate School of Veterinary Sciences, Gifu University, Gifu, Japan; 3 Department of Immunology and Microbiology, Pasteur Institute in Ho Chi Minh City, Ho Chi Minh City, Vietnam; 4 Department of Parasitology, College of Public Health, University of the Philippines Manila, Manila, Philippines; 5 Department of Biology, School of Science and Engineering, Ateneo de Manila University, Manila, Philippines; 6 Department of Immunopathology and Microbiology, College of Veterinary Medicine and Biomedical Sciences, Cavite State University, Cavite, Philippines; 7 Laboratory of Veterinary Pathology, Obihiro University of Agriculture and Veterinary Medicine, Hokkaido, Japan; 8 Department of Tropical Medicine and Parasitology, Dokkyo Medical University, Tochigi, Japan; 9 Graduate School of Agricultural and Life Sciences, The University of Tokyo, Tokyo, Japan

**Keywords:** *Schistosoma japonicum*, Biomarker, Peroxiredoxin-4, ELISA, Diagnosis

## Abstract

**Background:**

*Schistosoma japonicum*, which inhabits the mesenteric vein of the mammalian hosts for about 20 to 30 years, is subjected to the oxidative stresses from the host defense mechanism during their intra-mammalian stages. To counteract this host immune attack, the parasite utilizes their antioxidant system for survival inside the host. Peroxiredoxins (Prxs), thiol-specific antioxidant proteins, play an essential role for protecting the parasite against oxidative stress by reducing hydrogen peroxide to water. Only three types of 2-Cys Prxs have been previously characterized in *S. japonicum* whereas a fourth Prx has been identified for *Schistosoma mansoni* as Prx-4. A sequence coding homologous to this gene in the *S. japonicum* database was identified, characterized and expressed as recombinant SjPrx-4 protein (rSjPrx-4). Furthermore, rSjPrx-4 was evaluated in this study for its diagnostic potentials in detecting *S. japonicum* infection in humans.

**Results:**

The gene found in the parasite genome contained 2 active-site cysteines with conserved sequences in the predicted amino acid (AA) sequence and showed 75% identity with that of the previously characterized Prx (TPx-1) of *S. japonicum*. The gene was expressed in different stages of schistosome life-cycle with highest transcription level in the adult male. The gene was cloned into a plasmid vector and then transfected into *Escherichia coli* for expression of rSjPrx-4. Anti-rSjPrx-4 mouse sera recognized native SjPrx-4 in egg and adult worm lysate by western blotting. The result of a mixed function oxidation assay in which rSjPrx-4 prevented the nicking of DNA from hydroxyl radicals confirmed its antioxidant activity. Subsequently, immunolocalization analysis showed the localization of SjPrx-4 inside the egg, on the tegument and in the parenchyma of the adult worm. Enzyme-linked immunosorbent assay results showed that rSjPrx-4 has 83.3% sensitivity and 87.8% specificity. Its diagnostic potential was further evaluated in combination with recombinant SjTPx-1 protein, yielding an improved sensitivity and specificity of 90% and 92.7%, respectively.

**Conclusions:**

These results suggest that SjPrx-4 plays a role as an antioxidant dealing with oxidative stresses of *S. japonicum*, and its diagnostic potential improved by coupling it with SjTPx-1 is a proof for developing a serological test with better diagnostic performance for human schistosomiasis.
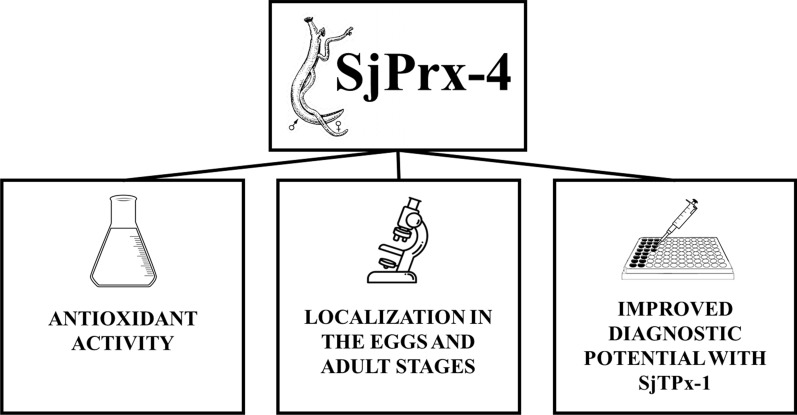

## Background

Schistosomiasis japonica, a zoonotic disease caused by *Schistosoma japonicum*, still remains as an economic and public health concern in China, the Philippines and some parts of Indonesia. A major consideration making the disease prevalence less controllable is the use of diagnostic methods with low efficiency in detecting infection and assessing the success of control measures [1]. The standard test for the diagnosis of intestinal schistosomiasis in endemic areas is still parasitological detection of eggs in stool samples such as Kato-Katz [2]. Although the Kato-Katz is simple and has a specificity of 100%, it is time-consuming, laborious, and poses unsatisfactory sensitivity mostly in endemic areas with low prevalence of schistosomiasis [3]. Therefore, many individuals with light to moderate intensity of infection were underdiagnosed using Kato-Katz method [4, 5].

Immunodiagnostic tools based on schistosome antigens have a high sensitivity and are less time consuming as compared to coprological diagnostic tools. Thus, these assays have become an attractive alternate for schistosomiasis surveillance especially in the locations where the disease has been nearly eliminated. Many antigens with high sensitivity and specificity have been reported as promising markers for diagnosing *S. japonicum* infection [6, 7]. However, single recombinant antigens used for the serodiagnosis of human schistosomiasis were found to have less sensitivity with lower *S. japonicum* infection intensity level in different human cohorts [8]. Employing multivalent cocktail antigens could possibly improve the low sensitivity of single recombinant antigens in leishmaniasis [9], in clonorchiais [10] and in malaria [11] as well as schistosomiasis [8]. Moreover, a cocktail-ELISA showed the improvement of specificities in humans, water buffaloes, and dogs for schistosomiasis japonica [12]. Therefore, identification of the synergistic diagnostic potentials of different combinations of specific antigens recognized by the host immune system is essential and will help improve the test on detecting the schistosome infection.

The schistosome adult worms can live in the host hepatic mesenteries for about 20–30 years without any treatment being administered to the host [13, 14]. In this environment, the parasite must deal with reactive oxygen species (ROS) mainly produced by the host immune system as well as those produced by the parasite itself during erythrocyte ingestion. To protect themselves, one of the defense mechanisms the parasite uses is through the production of an antioxidant firewall [15].

Peroxiredoxins (Prxs) are members of a family of antioxidant enzymes that detoxify hydrogen peroxide to water [16]. Prxs have been classified into 2-Cys Prx and 1-Cys Prx based on the number of cysteine residues involved in the peroxidase activity [17]. The 2-Cys Prxs are also known as thioredoxin peroxidases (TPx) because they use thiol from the thioredoxin system to carry out peroxidase function [18]. Three types of *S. japonicum* peroxiredoxin (Prx), also called thioredoxin peroxidase (TPx), have been cloned and characterized, namely TPx-1, TPx-2 and TPx-3 [19]. SjTPx-1 was found in the tegumental tissues of the adult worm and in excretory/secretory products [19, 20]. SjTPx-2 was mainly expressed in gut epithelium, vitelline gland, prarenchyma and in the sub-tegument of adult worm [19]. SjTPx-3 has mitochondrial signal and was considered to play a role in mitochondria [19, 21]. However, a fourth Prx, reported in *S. mansoni* as SmPrx-4 [22], has not yet been identified in *S. japonicum*.

Moreover, there have been many studies identifying thioredoxin peroxidases as potential antigens for diagnosing various parasitic diseases such as *Fasciola gigantica* infection in water buffaloes [23] and *Plasmodium falciparum* malaria in humans [24]. Among three types of *S. japonicum* thioredoxin peroxidase, SjTPx-2 and SjTPx-3 were evaluated as vaccine candidates against schistosome infection [21, 25] and only SjTPx-1 was identified as a promising candidate antigen for serodiagnosis of *S. japonicum* infection in humans [26], water buffaloes [27] and dogs [28].

In this study, the *S. japonicum* Prx-4 (SjPrx-4) gene was cloned and expressed. Then, its expression levels among different developmental stages and the antioxidant activity of the recombinant SjPrx-4 (rSjPrx-4) were further analyzed. Localization of the protein was also examined and its diagnostic potential for human schistosomiasis was evaluated as a single antigen and cocktail-antigen with SjTPx-1 using enzyme-linked immunosorbent assay (ELISA).

## Methods

### Parasites and animal infection

The *S. japonicum* Yamanashi strain was maintained using the snail intermediate host *Oncomelania hupensis nosophora*. Miracidia were collected after the eggs were hatched in fresh water. Then, *O. h. nosophora* snails were infected with 5 miracidia and were crushed 6 months later to collect the cercariae required in this study. Five female ICR mice aged 5 weeks-old (Clea Inc, Tokyo, Japan) were used for *S. japonicum* infection. The mice were percutaneously infected with 40 *S. japonicum* cercariae each and sacrificed at 8 weeks post-infection.

### Human sera

Archived serum samples collected from Filipinos living in Irosin, Sorsogon, Philippines (schistosomiasis endemic areas) in 2015 were used in this study in accordance with the ethical clearance approved by the University of the Philippines, Manila. Thirty schistosomiasis-positive and 41 schistosomiasis-negative serum samples were confirmed by Kato-Katz in triplicate slide examinations of approximately 41.7 mg fecal sample each and stool PCR for *S. japonicum* [26]. Moreover, 45 healthy American sera were used as negative controls (BioreclamationIVT, Baltimore, MD, USA). Archived sera from patients infected with *Paragonimus westermani* (*n* = 10), *Entamoeba histolytica* (*n* = 9), *Opisthorchis viverrini* (*n* = 6) and *Trichuris trichiura* (*n* = 1) were also used to test the cross-reactivity. *Entamoeba histolytica-*positive and *T. trichiura-*positive sera were collected from a schistosomiasis-free area in the Philippines and confirmed through microscopic examination. *Opisthorchis viverrini*-positive samples were taken from Thailand and *P. westermani*-positive sera were collected from Japanese patients diagnosed through either clinical manifestations or antibody detection [26].

### Sequence analysis

The coding sequence of SjPrx-4 (accession no. Sjp_0053380) was retrieved from GeneDB with the *S. mansoni* Prx-4 sequence as reference using BLASTX in NCBI (http://www.ncbi.nlm.nih.gov/). The Compute pI/Mw tool (http://www.Expasy. ch/tools/pi tool.html) was used to calculate the molecular weight and isoelectric point of SjPrx-4. The SignalP 4.0 was used to predict the signal peptide (http://www.cbs.dtu.dk/services/SignalP/).

### Preparation of rSjPrx-4

Primers of SjPrx-4 were designed according to the sequence obtained from GeneDB (accession no. Sjp_0053380) with restriction enzyme sites of *Bam*HI and *Xho*I (underlined) at the forward (5′-GCCG GAT CCA TGC TGT TAC CAA CG-3′) and reverse oligonucleotides (5′-GCCC TCG AGT CAT GTA GAT GAA GAG-3′), respectively. The PCR product was directly cloned into pCR 2.1-TOPO vector (Invitrogen, CA, USA) according to the manufacturer’s protocol. And the plasmids were transformed into *Escherichia coli* Mach I (Invitrogen). Selected clones were confirmed by sequencing for the sequence identity. Then the SjPrx-4 gene was digested with *Bam*HI and *Xho*I to be subcloned from pCR 2.1-TOPO vector into pGEX-6P-1 vectors (Novagen, Madison, USA). Subsequently, pGEX-6P-1 plasmids containing the SjPrx-4 gene were transfected into *E. coli* strain Rosetta (DE3) (Novagen, MA, USA). The expression of rSjPrx-4 was induced by 1 mM isopropyl-β-D-thiogalactoside (IPTG) at 25 °C overnight. The bacteria pellets were collected by centrifuging at 10,000×*g*, 4 °C for 20 min and were sonicated in lysis buffer. Cellular debris was pelleted at 8000×*g*, 4 °C for 20 min. The supernatant was collected and purified using glutathione Sepharose 4B resin and cleaved by PreScission Protease to remove glutathione S-transferase (GST)-tag (GE Healthcare BioSciences AB, Uppsala, Sweden) following the manufacturer’s instructions. Purified rSjPrx-4 protein was then dialyzed 3 times with phosphate-buffered saline (PBS), pH 7.2. The concentration of the rSjPrx-4 was determined by bicinchoninic acid (BCA) assay (Thermo Fisher Scientific Inc., Rockford, IL, USA), and the protein was stored at − 80 °C until use.

### Anti-rSjPrx-4 serum development

To obtain anti-rSjPrx-4 antibodies, rSjPrx-4 emulsified with the TiterMax Gold® adjuvant (Sigma-Aldrich, St. Louis, MO, USA) was inoculated subcutaneously into 8 week-old female ICR mice (100 µg rSjPrx-4/200 μl inoculum/mouse). Five mice were immunized thrice with a 2-week interval by subcutaneous injection. Then mouse sera were collected, pooled and labeled as anti-rSjPrx-4 mouse serum after the 3rd immunization. The anti-rSjPrx-4 mouse serum was used for immunohistochemistry analysis as primary antibody.

### Quantitative real time reverse transcription-PCR (qRT-PCR) analysis

The transcription level of the SjPrx-4 gene in each developmental stage was examined by qRT-PCR in triplicates. The intramolluscan stages of *S. japonicum* were obtained from schistosome-infected *O. h. nosophora* whereas the intramammalian stages were from infected mice. The total RNA samples were prepared from adult worm, egg and sporocyst with TRIZOL reagent (Invitrogen, CA, USA) according to the manufacturer’s instruction. The PrimeScript RT reagent kit (Takara, Shiga, Japan) was used to synthesize the cDNA library. The forward (5′-GTT ACC AAC GAA ACC GGC G-3′) and reverse (5′-CCC AAT CCT CCT GCT TTA CGA-3′) primers were used to amplify the SjPrx-4 gene with a size of 273 bp. Triose-phosphate isomerase (TPI) gene (forward primer: 5′-ATG GCA GTA GAG CCG ACA AC-3′; reverse primer: 5′-AAC GCT TAG ACC TTC TGC AA-3′) was used as an internal standard gene [19]. Twenty microliters of PCR reaction mixture was prepared containing 0.5 µl of template cDNA, 10 µl of PowerUp SYBR Green Master Mix (Applied Biosystems, UAB, Lithuania), 200 nM of each primer, and distilled water up to 20 µl. The reactions were run on an Applied Biosystems 7900HT instrument (Applied Biosystems, Foster City, CA, USA) under the following conditions: 95 °C for 3 min, 40 cycles at 95 °C for 30 s and at 60 °C for 1 min, and a dissociation stage as per the instrument guidelines. The 2^−ΔΔCq^ method [29, 30] was performed to analyze data with TPI gene employed as the internal control. The transcriptional changes of SjPrx-4 gene in the eggs, male and female adult worms were calculated relative to that of the sporocyst. Negative controls not containing the parasite DNA were included in each PCR run.

### Antioxidant activity of rSjPrx-4

A mixed-function oxidation (MFO) assay was performed to evaluate the antioxidant activity of rSjPrx-4 [31]. The mixture of 50 µl containing 25 mM HEPES (pH 7.0), 10 mM dithiothreitol (DTT), 20 mM EDTA and 40 µM FeCl_3_, was pre-incubated without or with rSjPrx-4 (10 ng, 100 ng, 1 µg and 5 µg/ml) for 1 h at 37 °C. After pre-incubation 500 ng of supercoiled pBluescript plasmid DNA (Stratagene, La Jolla, CA, USA) was added into the reaction mixture and incubated for another 3 h at 37 °C. Finally, 0.8% agarose gel electrophoresis was used to assess the nicking of the supercoiled plasmid by MFO. The heat-denatured rSjPrx-4 protein was included as negative control.

### Western blotting

The specific binding of anti-rSjPrx-4 with SjPrx-4 and the expression of SjPrx-4 in *S. japonicum* eggs and adult worms were determined by western blotting. The egg and adult worm lysate were analyzed on 12% SDS-PAGE and transferred onto a polyvinylidene difluoride (PVDF) membrane. The membrane was blocked with 3% skimmed milk in Tris-buffered saline with 0.05% of Tween 20 (TBS-T) overnight at 4 °C. Then, the membrane was incubated with anti-rSjPrx-4 at 1:100 dilutions in blocking buffer for 1 h at room temperature (RT). After washing 3 times with TBS-T, the membrane was further incubated with an anti-mouse IgG conjugated with horseradish peroxidase (HRP) (GE Healthcare) at dilution of 1:5000 for 1 h at RT. After washing, specific binding of anti-rSjPrx-4 was visualized using SuperSignal HRP chemiluminescent substrates (ThermoFisher Scientific) and an ImageQuant LAS 500 chemiluminescence detection machine (GE Electric, Tokyo, Japan). Membranes containing egg and adult lysate treated with normal mouse serum were used as negative controls, separately.

### Immunolocalization of SjPrx-4 in schistosomes

Immunohistochemistry was performed on 10% formalin-fixed paraffin embedded sections of adult worms and visceral organs obtained from the infected mice according to standard techniques [32], with some modifications. The heat-induced antigen retrieval method was done with microwave at 97 °C in citrate buffer (pH 6.0) for 15 min. Inhibition of endogenous peroxidase activity was performed by incubating the slides with 0.3% hydrogen peroxide at RT for 10 min. The sections on the slide glasses were incubated with the anti-SjPrx-4 mouse serum (1:200 in PBS) at 4 °C overnight in a humidified chamber. Non-specific antigen-antibody reaction was blocked with Histofine Mouse Stain Kit (Nichirei Biosciences, Tokyo, Japan) whereas Histofine Simplestain AP (Nichirei Biosciences) was used as the secondary antibody, both according to the manufacturer’s instructions. Labeling was visualized with a Fast Red chromogen (Fast Red II Substrate Kit; Nichirei Biosciences), and the sections were counterstained with Meyer’s hematoxylin. The sections incubated with normal mouse serum instead of the anti-SjPrx-4 mouse serum were used as negative controls.

### ELISA with recombinant antigens

ELISAs were performed with rSjPrx-4, rSjTPx-1 and combination of rSjPrx-4 and rSjTPx-1 (mixed in equivalent molar) using a panel of 45 negative control samples from non-endemic USA, 41 schistosomiasis-negative and 30 schistosomiasis-positive samples confirmed through Kato-Katz from endemic municipalities in the Philippines. Sera from other parasitic infections, i.e. with *P. westermani*, *E. histolytica*, *O. viverrini*, *T. trichiura*, were used to check the cross-reactivity with the combination of rSjPrx-4/rSjTPx-1. Two hundred ng/well of the recombinant antigens or *S. japonicum* soluble egg antigen (SEA) diluted with carbonate/bicarbonate buffer (pH 9.6) were coated in the microplate plates (ThermoFisher Scientific, Roskilde, Denmark) overnight at 4 °C. After 3 washes with PBS containing 0.05% Tween 20 (PBS-T) (Wako, Osaka, Japan), blocking was performed with 120 µl of 3% skimmed milk in PBS-T at RT for 5 min. Then, 100 µl of serum samples at a dilution of 1:400 in blocking buffer was added to each well in triplicate and incubated for 1 h at 37 °C. After washing, the plates were incubated with 100 μl of HRP-conjugated anti-human IgG (1: 20,000) (Proteintech, Manchester, UK) as the secondary antibody at 37 °C for 1 h. Subsequently, the plates were washed 3 times with T-PBS and incubated with 100 µl of HRP substrate, TMB (KPL, Gaithersburg, MD, USA) for 10 min. Fifty microliters of 1 M phosphoric acid was used to stop the reaction. The absorbance was measured at 450 nm using a microplate reader (MTP-500; Corona Electric, Tokyo, Japan). The cut-off OD values were calculated from the values of 45 non-endemic controls as mean + 3SD.

### Statistical analysis

Data from qRT-PCR were represented as the mean ± standard error (SE). Student’s t-test was used to analyze data, with a *P*-value less than 0.05 considered significant. The online software MedCalc (https://www.medcalc.org/calc/diagnostic_test.php) was used to compute the sensitivity and specificity of the ELISA tests.

## Results

### Cloning, expression and purification of rSjPrx-4

**Fig. 1:**
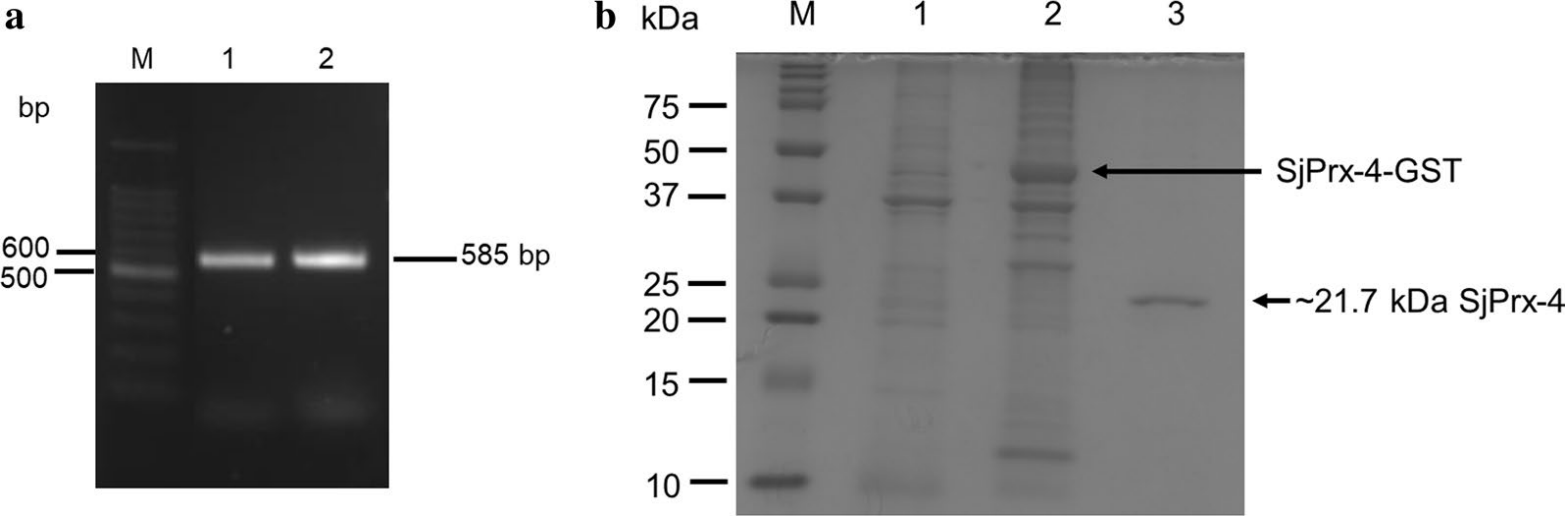
Gel electrophoresis and SDS-PAGE of SjPrx-4. **a** Agarose gel electrophoresis image of the PCR-amplified SjPrx-4 gene from the cDNA library of eggs and adult worms. Lane M: marker; Lane 1: eggs; Lane 2: adult worms. **b** SDS-PAGE of recombinant SjPrx-4. rSjPrx-4 was expressed in *E. coli*. Lane M: marker; Lane 1: *E. coli* lysate before adding IPTG; Lane 2: *E. coli* lysate at 25 h after adding IPTG shows expression of the recombinant protein with GST-tag (SjPrx-4-GST); Lane 3: purified rSjPrx-4 without the GST-tag (SjPrx-4)

The full-length coding sequence of SjPrx-4 was obtained by RT-PCR from the cDNA libraries of eggs and 8-week-old adult worms (Fig. 1a). The SjPrx-4 gene was 585 bp in size which was identical to the one described in GeneDB and coded a protein of 194 amino acids with a predicted molecular weight of 21.7 kDa and an isoelectric point of 7.6. The deduced polypeptide sequence of SjPrx-4 was 93.3% identical to its homologous gene SmPrx-4 in *S. mansoni* (GenBank: XP_018651610.1) and 59.3–75.0% identity with SjTPx-3 (GenBank: BAD90103.1), SjTPx-2 (GenBank: BAD90102.1) and SjTPx-1 (GenBank: BAD01572.1) (Additional file 1: Table S1, Additional file 2: Figure S1). SjPrx-4 contained 2 cysteine conserved sequences with the peroxidatic cysteine residue at the N-terminal FYPADFTFVCPTE and the resolving cysteine residue at C-terminal GEVCPA as active sites (Additional file 1: Table S1, Additional file 2: Figure S1). The coding sequence of SjPrx-4 was successfully expressed as a GST fusion protein in *E. coli* Rosetta (DE3) (Fig. 1b, Lane 2). SDS-PAGE showed rSjPrx-4 with an expected size of 21.7 kDa after removing the GST tag (Fig. 1b, Lane 3).

### Transcription levels of SjPrx-4 gene at different developmental stages of *S. japonicum*

**Fig. 2:**
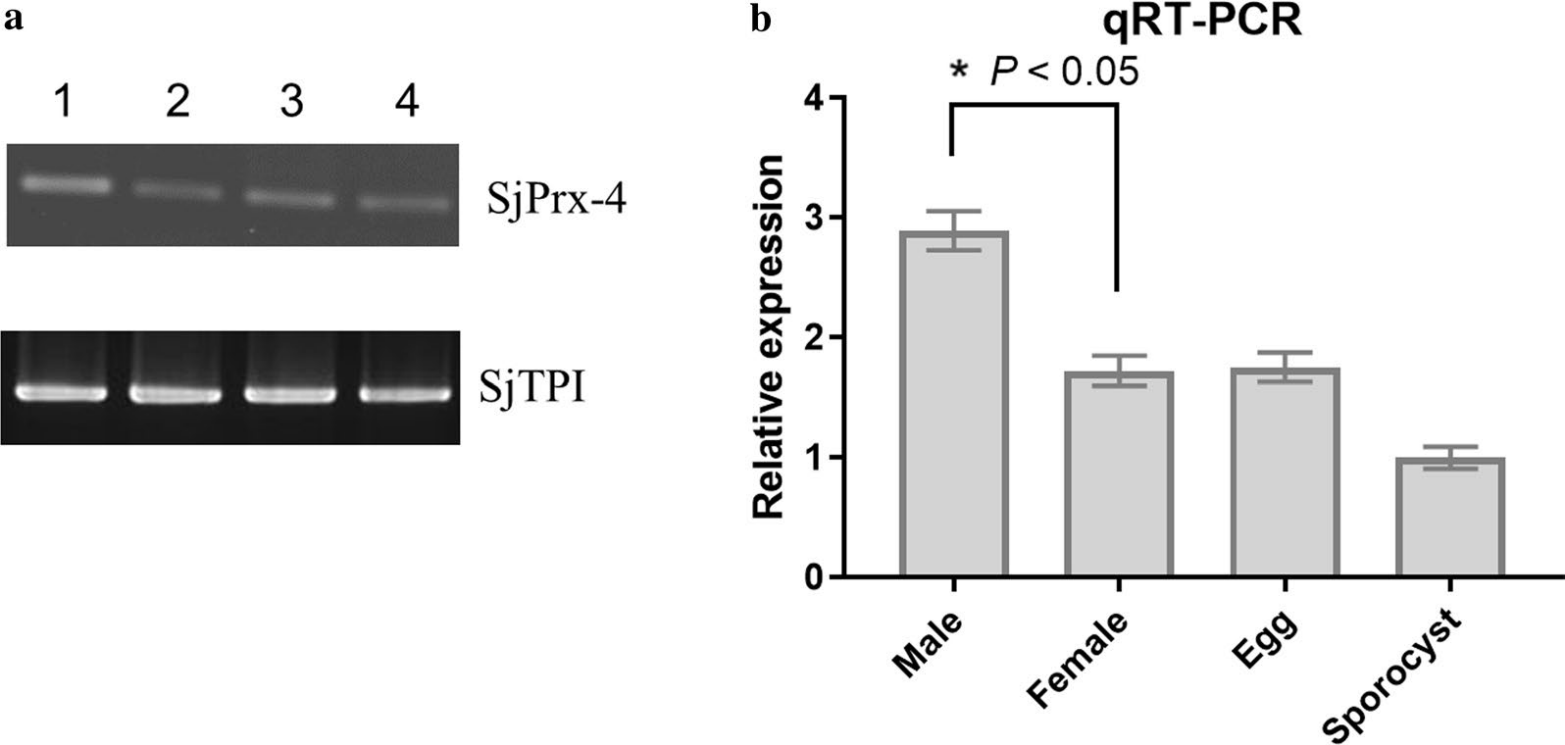
Different expression profiles of the SjPrx-4 gene at various developmental stages of *S. japonicum*. **a** Transcription of the SjPrx-4 gene was detected by RT-PCR in the different developmental stages. Lane 1: male; Lane 2: female; Lane 3: eggs; Lane 4: sporocysts. Upper panel: transcription of SjPrx-4 gene; Lower panel: transcription of SjTPI gene as internal control. **b** Expression levels of the SjPrx-4 gene were determined by qRT-PCR. Data were analyzed according to 2^−ΔΔCq^ method using the TPI gene as the internal control for each sample. The fold changes of transcription levels in eggs and male and female worms were calculated relative to that of the sporocysts. Data are represented as the mean ± SE of three independent assays. *P < 0.05

RT-qPCR was performed to examine the transcription levels of SjPrx-4 in eggs, sporocysts and 8-week-old male and female adult worms. SjPrx-4 transcript was found in all stages examined (Fig. 2a). SjPrx-4 transcription expression was highest level in males, followed by eggs and females (Fig. 2b). On the other hand, sporocysts showed the lowest transcription level of SjPrx-4 (Fig. 2b).

### Antioxidant activity of rSjPrx-4

**Fig. 3:**
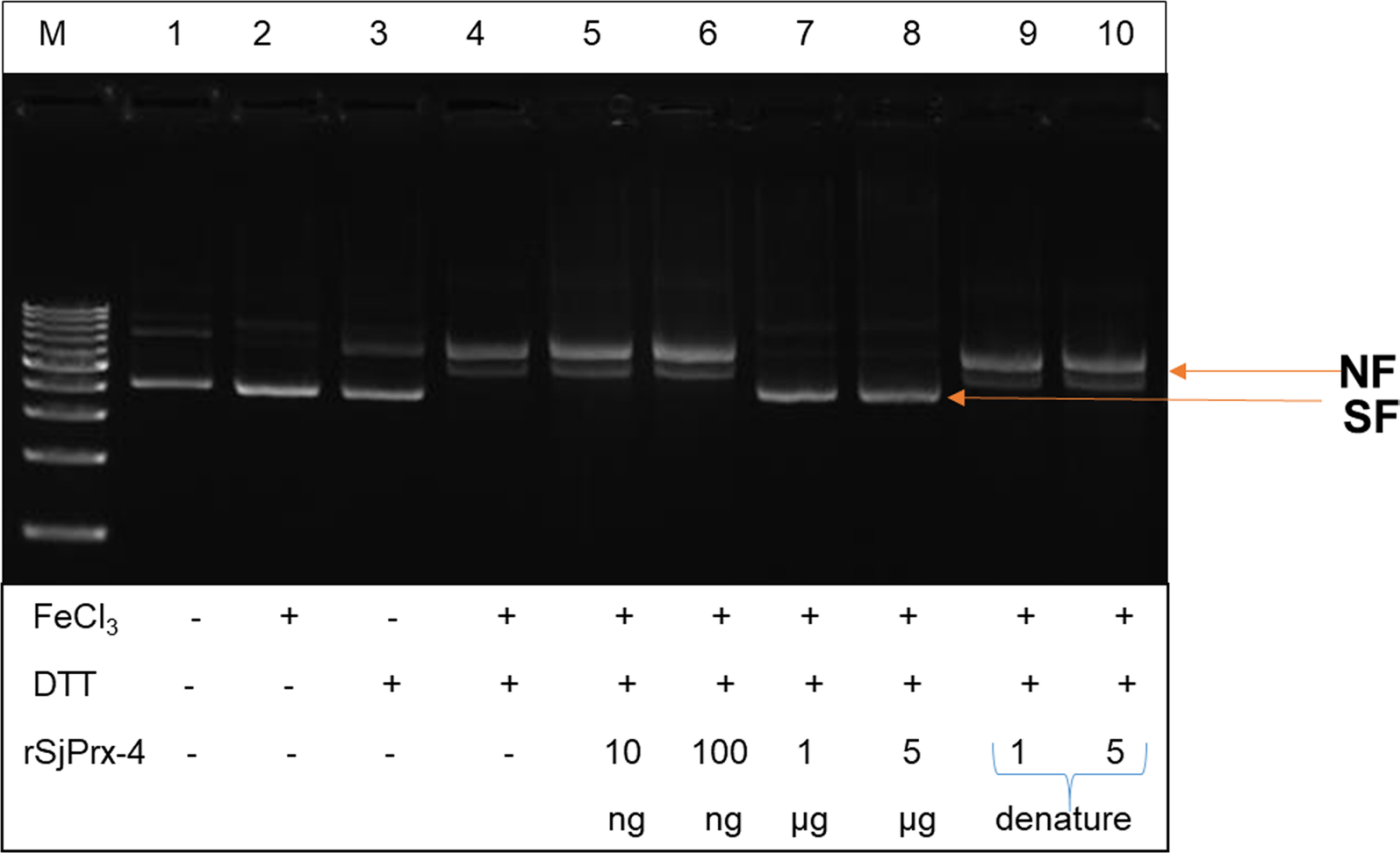
Antioxidant activity of SjPrx-4. Lane 1: pBluescript plasmid DNA; Lane 2: pBluescript plasmid DNA+ FeCl_3_; Lane 3: pBluescript plasmid DNA+DTT; Lane 4: pBluescript plasmid DNA + FeCl_3_ + DTT; Lanes 5–8: pBluescript plasmid DNA + FeCl_3_ + DTT and 10 ng, 100 ng, 1 µg, and 5 µg/ml of rSjPrx-4, respectively; Lanes 9, 10: pBluescript plasmid DNA + FeCl_3_ + DTT and 1 µg, and 5 µg/ml of denatured rSjPrx-4, respectively. The nicked form (NF) and supercoiled form (SF) are indicated on the right

The antioxidant activity of rSjPrx-4 was measured as the capacity in protection of supercoiled DNA from nicking by using the MFO system. The MFO system generated hydroxyl radicals that changed Bluescript supercoiled plasmid DNA to nicked form as confirmed by its increased molecular weight in the agarose gel (Fig. 3, Lane 4). However, the presence of rSjPrx-4 at concentrations of above 1 µg/ml prevented the nicking of supercoiled plasmid DNA (Fig. 3, Lanes 7, 8). In addition, presence of heat-inactivated rSjPrx-4 did not prevent the nicking of supercoiled plasmid DNA (Fig. 3, lane 9, 10). These results confirmed the antioxidant activity of rSjPrx-4.

### Western blot analysis of anti-rSjPrx-4 against egg and adult worm lysates

**Fig. 4:**
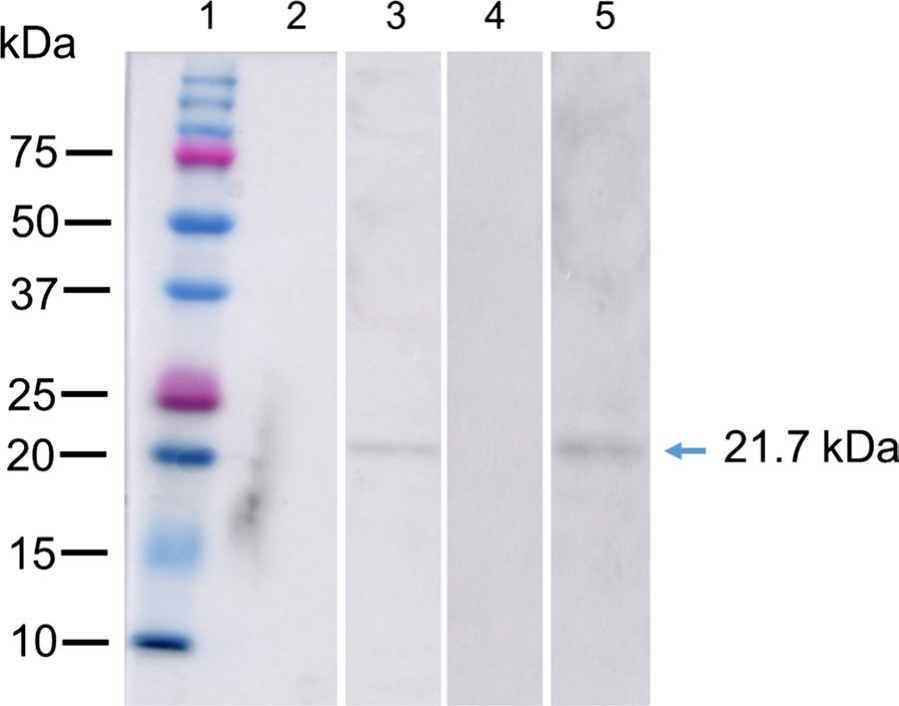
Western blot analysis of *S. japonicum* egg and adult worm lysates with anti-SjPrx-4 mouse sera. Lane 1: protein marker; Lane 2: *S. japonicum* egg lysate incubated with normal mouse sera; Lane 3: *S. japonicum* egg lysate incubated with anti-SjPrx-4 mouse sera; Lane 4: adult worm lysate incubated with normal mouse sera; Lane 5: adult worm lysate incubated with anti-SjPrx-4 mouse sera

Anti-rSjPrx-4 mouse serum was used to detect native SjPrx-4 in eggs and adult worms. The results showed the presence of a band in egg and adult worm lysates at approximately 21.7 kDa as the expected size of the protein (Fig. 4, Lanes 3, 5). The normal mouse serum showed no specific bands with the protein extract of egg and adult worms (Fig. 4, Lanes 2, 4).

### Immunolocalization of SjPrx-4 in *S. japonicum* eggs and adult worms

**Fig. 5:**
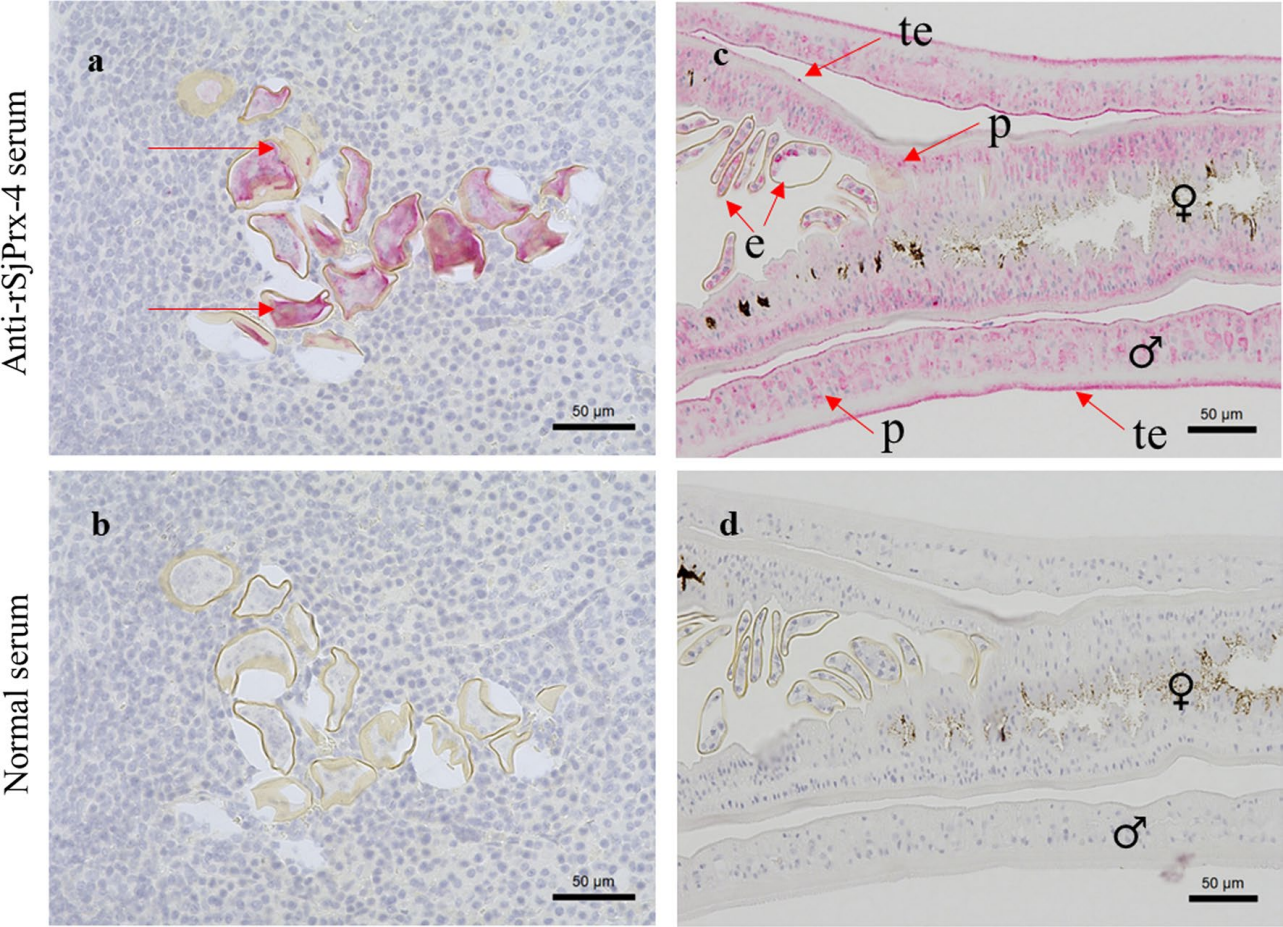
Immunolocalization of SjPrx-4 in eggs and adult worms of *S. japonicum*. The red arrows indicate the existence of SjPrx-4 expression. **a** The liver section from *S. japonicum*-infected mice reacted with anti-SjPrx-4 mouse serum shows immunopositivity in the space between the miracidium and the egg-shell. **b** There is no reaction in the liver section from *S. japonicum*-infected mice reacted with normal mouse serum. **c** Immunopositive reactions of SjPrx-4 were observed in the tegument (te) and in the parenchyma (p) of *S. japonicum* adult worms (♀: female and ♂: male). **d** The section from *S. japonicum* adult worms reacted with normal mouse serum did not reveal any immunopositive reactions. *Scale-bars*: 50 µm

Immunohistochemical staining was conducted to observe the localization of SjPrx-4 in the eggs deposited in the infected liver sections and schistosome adult worms, using the mouse anti-rSjPrx-4 serum. Sections treated with the anti-rSjPrx-4 serum showed that SjPrx-4 was mainly localized inside the egg and in the parenchyma as well as on the tegument of adult male worms (Fig. 5a, c), as opposed to the sections incubated with normal mouse serum, which showed no positive reactions (Fig. 5b, d).

### ELISA with rSjPrx-4, rSjTPx-1 and a combination of SjPrx-4/SjTPx-1

**Fig. 6:**
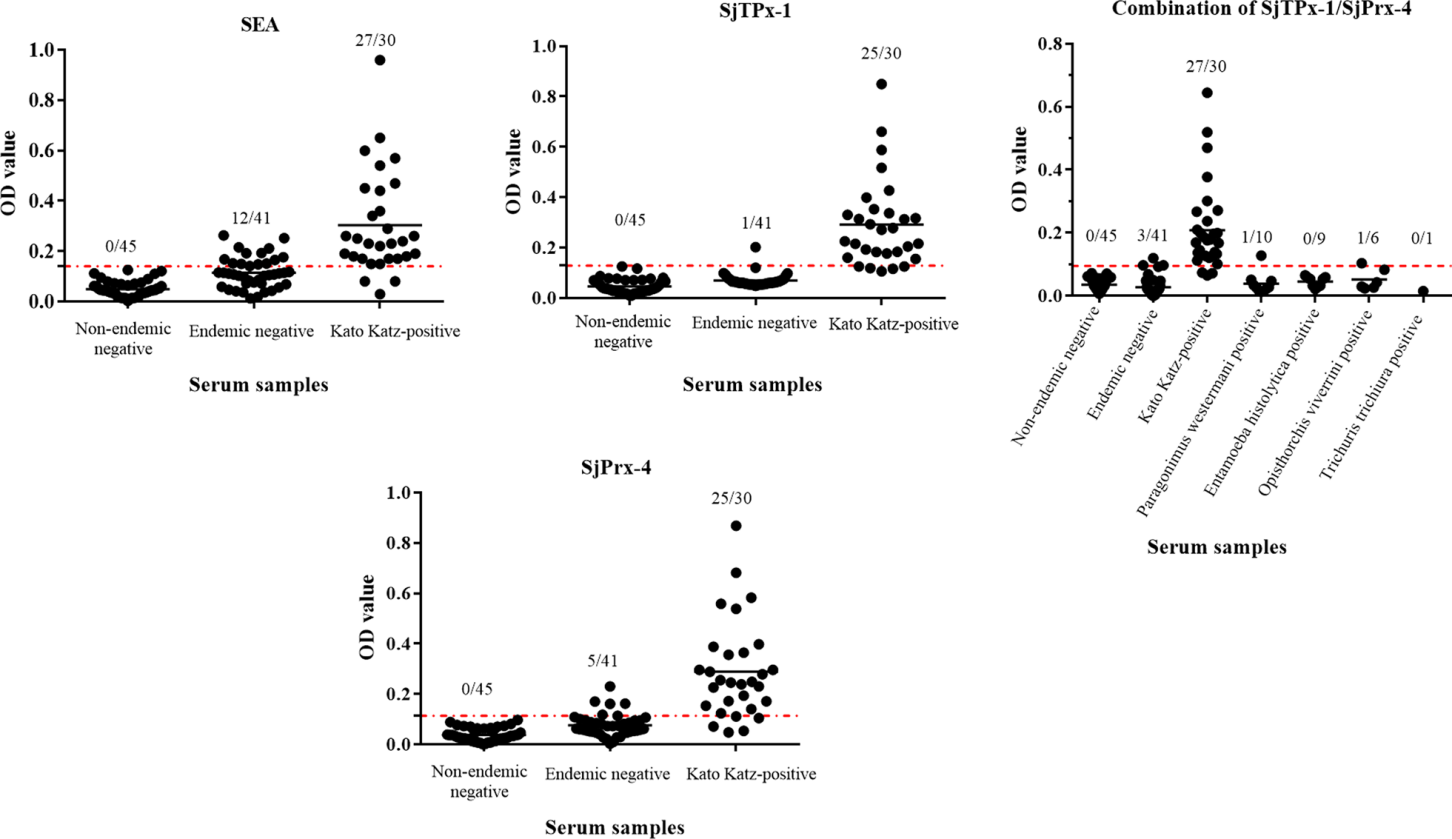
ELISA results with SjPrx-4, SjTPx-1 recombinant antigens and a combination of rSjPrx-4/rSjTPx-1. Cut-off values are represented as the mean + 3SD values of 45 non-endemic controls by the red dotted lines. The number of samples having OD values higher than the cut-off values are shown

SEA detected 27 out of 30 schistosomiasis-confirmed sera (90.0%) as positive and 29 out of 41 non-endemic sera (70.7%) as negative (Fig. 6, Additional file 3: Table S2). Individually, the SjPrx-4-ELISA showed 83.3% sensitivity and 87.6% specificity whereas the SjTPx-1-ELISA had 83.3% sensitivity and 97.6% specificity (Fig. 6, Additional file 3: Table S2). However, when rSjTPx-1 and rSjPrx-4 were combined as ELISA antigens, the sensitivity and specificity of the test improved to 90.0% and 92.7%, respectively (Fig. 6, Additional file 3: Table S2). Moreover, the results of combined SjTPx-1/SjPrx-4 ELISA analyzed by receiver-operating characteristic curves showed the best sensitivity of 97.6% and specificity of 90.0% (Additional file 4: Figure S2).

Human serum samples collected from the patients infected with *T. trichiura* and *E. histolytica* had no cross-reaction in the ELISA with a combination of rSjPrx-4 and rSjTPx-1. However, 1 out of 6 *O. viverrini-*infected and 1 out of 10 *P. westermani-*infected human sera showed minimum cross-reaction against the combined antigen with OD values slightly higher than the cut-off value.

## Discussion

Molecular cloning and characterization of 2-Cys peroxiredoxins such as SjTPx-1, SjTPx-2 and SjTPx-3 from *S. japonicum* [19, 21, 25] as well as SmTPx-1 [33], SmTPx-2 and SmTPx-3 [34] from *S. mansoni* have been reported in previous research. A fourth type, SmPrx-4, was first identified by Protasio et al. [22] in 2012 when they systematically improved the *S. mansoni* draft genome using a combination of Sanger and Next Generation, sequencing. Moreover, the Prx-4 homologue was also found in *S. mekongi* when egg proteins were analyzed using proteomic strategies [35]. In the present study, a novel Prx-4 homologue from *S. japonicum* (SjPrx-4) was successfully cloned and characterized.

We have obtained the SjPrx-4 complete sequence comprised of 585 bp that encoded 194 amino acids without a signal peptide and with a predicted molecular weight of 21.7 kDa. Bioinformatics analysis showed that SjPrx-4 was most closely related to SmPrx-4 exhibiting two conserved cysteines, the peroxidatic Cys and the resolving Cys. These cysteines of the 2-Cys Prx family are known to be essential in Cys-dependent thioredoxin peroxidase activity [36]. Moreover, the amino acid sequence of SjPrx-4, SjTPx-1 and SjTPx-2 contains the GVL motif, which was reported to be essential to the catalase-like activity of Prx-1 as seen in the green spotted puffer fish *Tetradon nigroviridis* [37]. The catalase-like activity might explain how some parasitic helminths such as *Fasciola hepatica* and *S. mansoni* are deficient in catalase [38] and have relatively low levels of glutathione peroxidase [39], but possess highly expressed Prxs [40, 41]. SjPrx-4 as well as SjTPx-1 and SjTPx-2 may be enzymes having dual-functions by acting as Cys-dependent thioredoxin peroxidase and having a catalase-like activity at the same time. The catalase-like activity of SjPrx-4, SjTPx-1 and SjTPx-2 should however be confirmed with further studies.

The results of antioxidant activity showed Prx proteins from other helminths had similar activity in protecting supercoiled plasmid DNA in a MFO assay [34, 42]. In this study, rSjPrx-4 also showed antioxidant activity in protection of pBluescript from supercoiled DNA form to nicked form in MFO system. This finding suggested that SjPrx-4 might help the parasite to neutralize the ROS generated from the host immune system as well as from the internal metabolism of worms themselves.

The RT-qPCR results showed the presence of SjPrx-4 in all the stages of *S. japonicum* examined here. The finding that the expression of the SjPrx-4 transcript in male worms was higher than that of female worms may imply the fact that the latter require less SjPrx-4 expression since they are enclosed in the gynecophoral canal of the male worms. The present study showed that expression profile of SjPrx-4 was different from the three other TPxs. In contrast, RT-qPCR results of SjTPx-1, SjTPx-2 and SjTPx-3 showed that their mRNA in adult female worms were more abundant than in adult male worms. It was suggested that adult female worms may express the three other TPxs at a higher level than adult male worms [21, 25]. The result of RT-qPCR of SjPrx-4 was also supported by the immunohistochemistry experiments in the present study, showing that SjPrx-4 was mainly localized in the tegument of adult male worms as compared to the adult female worms. The more exposed male worms in turn express high levels of this enzyme to protect themselves from oxidative stress posed by the host immune system.

Immunolocalization of SjPrx-4 revealed that the protein is distributed inside the egg-shell, in the parenchyma and in the tegument of the adult worm. These results are different from the other reported *S. japonicum* Prxs: SjTPx-1 is a tegumental and circulating antigen; SjTPx-2 exists in the sub-tegument, parenchyma, gut epithelia and vitelline gland; and SjTPx-3 is a tegumental antigen having a mitochondrial signal peptide [19, 21]. It was suggested that the antioxidant functions of TPx-2 are associated with intracellular redox signaling [19] whereas those of SjTPx-3 are associated with the mitochondria [19, 21, 43]. The Prx family may have different roles at different locations and compensate with each other to protect the parasite from oxidative stresses. SjPrx-4 may have a chance to be exposed to the host’s immune system during infection, and thus may be a potential antigen for serodiagnosis in detecting the parasite infection.

The western blot results were consistent with those of immunolocalization and RT-qPCR in the egg and adult worm stages. The appearance of a single band showed that anti-SjPrx-4 can specifically recognize the native SjPrx-4 of *S. japonicum*.

The rSjPrx-4 and rSjTPx-1 used as a single antigen in the ELISA showed a positive rate of 83.3% in comparison with the 90% sensitivity of SEA. However, the specificity of rSjPrx-4 and SjTPx-1 was higher (at 87.8% and 97.6%, respectively) than that of SEA at 70.7%. Although both rSjPrx-4 and rSjTPx-1 were shown to be potential diagnostic antigens, their sensitivity and specificity need to be further improved to meet the current needs of schistosomiasis diagnosis. A way to improve the diagnostic capabilities of ELISA in parasitic diseases is combining multiple antigens in a cocktail format. Some of the patient sera have high OD values with SjPrx-4 but not with SjTPx-1 and *vice versa* (Additional file 5: Figure S3). Hence, two antigens might complement each other to have a better synergistic diagnostic potential. The combination of SjTPx-1 and SjPrx-4 antigens has improved the sensitivity of the ELISA to 90.0%. There have already been some reports on the application of multi-epitope recombinant antigens (chimeric proteins) to improve the sensitivity of serodiagnostic tests for schistosomiasis in experimental animals, goats and water buffaloes [44–46]. Therefore, it appears possible to construct multi-epitope recombinant antigens of SjTPx-1 and SjPrx-4 to enhance the accuracy and reliability of the diagnostic tools in detecting human schistosomiasis.

## Conclusions

Our study suggests that SjPrx-4 may play a role as an antioxidant of *S. japonicum* to deal with oxidative stress and that rSjPrx-4 could be a potential antigen for use in ELISA-based diagnosis of *S. japonicum* infection in humans. The combination of SjPrx-4 and SjTPx-1 as sensitive complementary diagnostic antigens suggests its use in the development of fusion antigens that could improve the sensitivity of ELISA assays in detecting human *S. japonicum* infection.

## Supplementary information

**Additional file 1: Table S1.** Identity of the SjPrx-4 amino acid sequence with SmPrx-4, SjTPx-1, SjTPx-2 and SjTPx-3.

**Additional file 2: Figure S1.** Amino acid sequence alignment of SjPrx-4 with SmPrx-4, SjTPx-1, SjTPx-2 and SjTPx-3.

**Additional file 3: Table S2.** Statistical analysis of ELISA results of rSjTpx-1, rSjPrx-4 and a combination of rSjPrx-4/rSjTPx-1.

**Additional file 4: Figure S2.** Receiver operating characteristic (ROC) curves for ELISA of SEA, SjPrx-4, SjTPx-1 and combination of SjTPx-1/SjPrx-4 against sera from endemic negative (*n* = 41) and schistosomiasis-positive by Kato-Katz (*n* = 30).

**Additional file 5: Figure S3.** Kinetics of 30 *S. japonicum* stool-positive sera response to SjTPx-1 and SjPrx-4.

## Data Availability

Data supporting the conclusions of this article are included within the article and its additional files.

## Abbreviations

Cq: threshold cycle
ELISA: enzyme-linked immunosorbent assay
GST: glutathione S-transferase
HRP: horseradish peroxidase
ICR: Institute of Cancer Research
IPTG: isopropyl-β-D-thiogalactoside
MFO: mixed-function oxidation
Prx: peroxiredoxin
PVDF: polyvinylidene difluoride
qRT-PCR: quantitative real time polymerase chain reaction
SE: standard error
SDS-PAGE: sodium dodecyl sulfate polyacrylamide gel electrophoresis
TBS-T: tris-buffered saline containing 0.05% Tween 20
TPI: triose-phophate isomerase
TPx: thioredoxin peroxidase
PBS-T: phosphate-buffered saline containing 0.05% Tween 20
