# Supplementary material for: Analyses of the expression, immunohistochemical properties and serodiagnostic potential of Schistosoma japonicum peroxiredoxin-4
Source: Parasit Vectors. 2020 Sep 1;13:436. doi: 10.1186/s13071-020-04313-w (PMC7460784; doi:10.1186/s13071-020-04313-w)
Supplement: Supplementary file 1 — Additional file 1: Table S1. Identity of the SjPrx-4 amino acid sequence with SmPrx-4, SjTPx-1, SjTPx-2 and SjTPx-3. [file 13071_2020_4313_MOESM1_ESM.docx]

|  | Number of SjPrx-4 amino acids match in length | % identity |
| --- | --- | --- |
| SmPrx-4 aa | 181/194 | 93.3 |
| SjTPx-1 aa | 138/184 | 75.0 |
| SjTPx-2 aa | 133/194 | 68.6 |
| SjTPx-3 aa | 115/194 | 59.3 |

**Additional file 1: Table S1.** Identity of SjPrx-4 amino acid sequence with SmPrx-4, SjTPx-1, SjTPx-2 and SjTPx-3
