# Supplementary figures and images for: Analyses of the expression, immunohistochemical properties and serodiagnostic potential of Schistosoma japonicum peroxiredoxin-4
Source: Parasit Vectors. 2020 Sep 1;13:436. doi: 10.1186/s13071-020-04313-w (PMC7460784; doi:10.1186/s13071-020-04313-w)

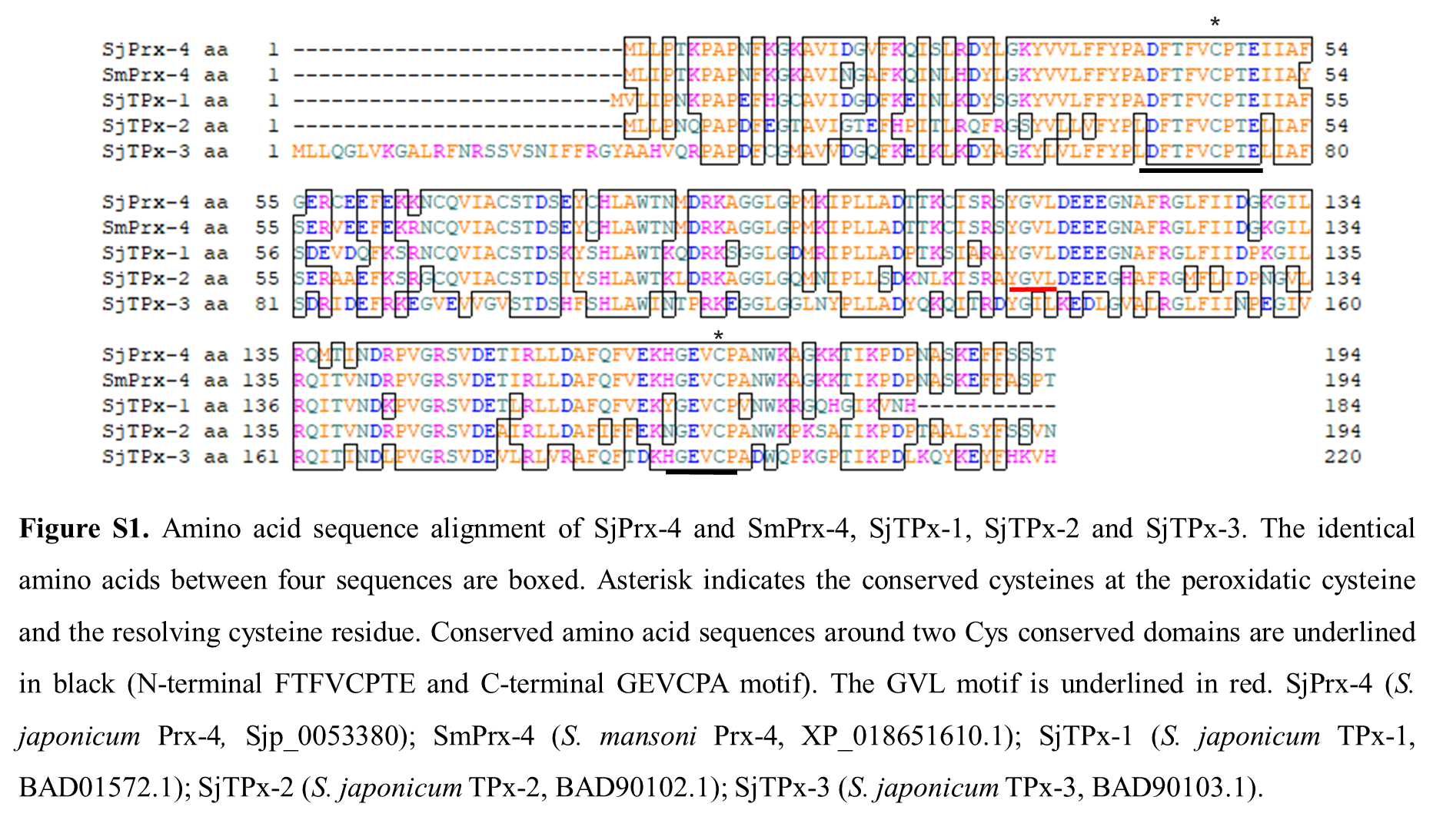

Supplement: Supplementary file 2 — Additional file 2: Figure S1. Amino acid sequence alignment of SjPrx-4 with SmPrx-4, SjTPx-1, SjTPx-2 and SjTPx-3. [file 13071_2020_4313_MOESM2_ESM.tiff]

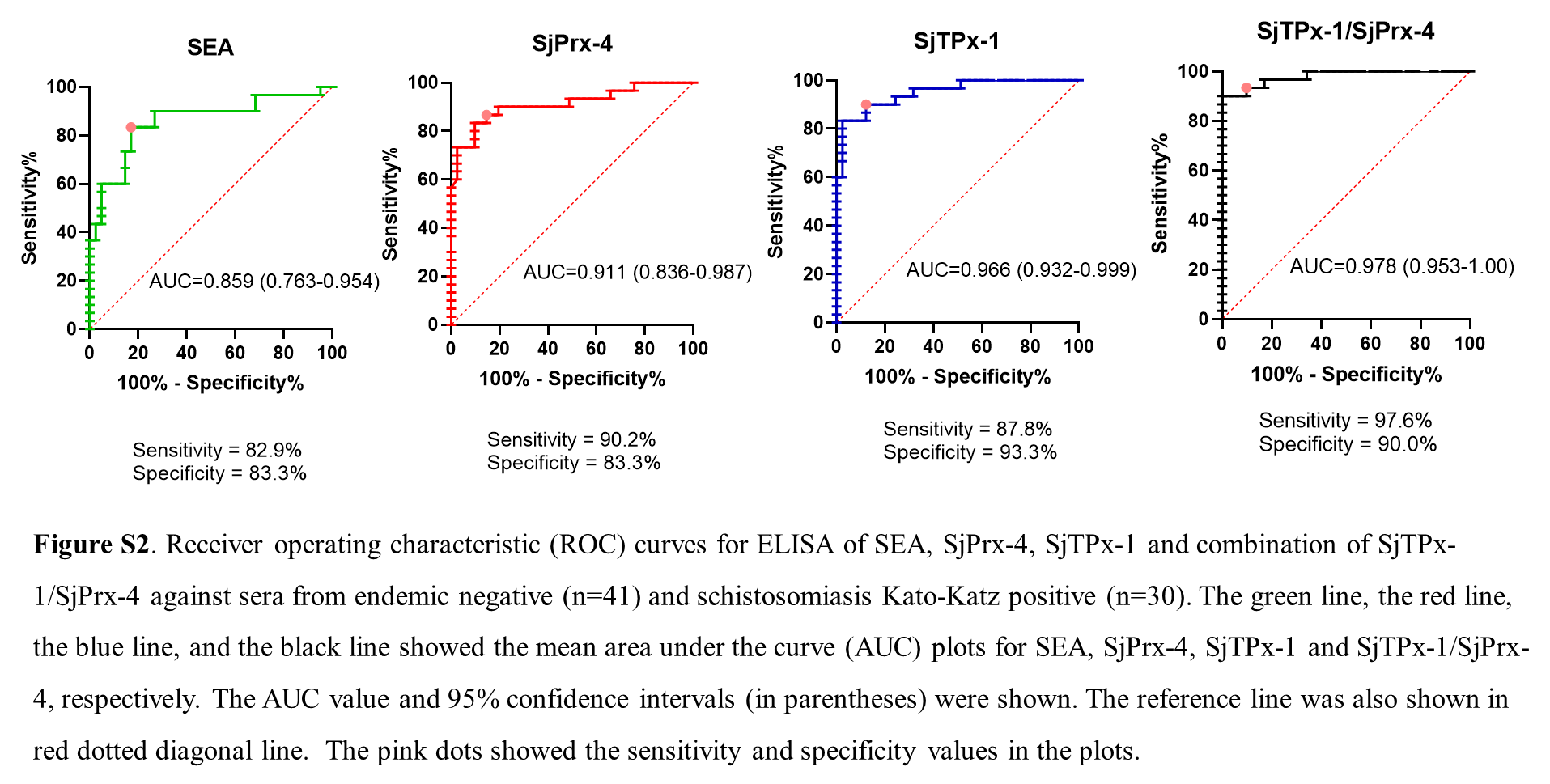

Supplement: Supplementary file 4 — Additional file 4: Figure S2. Receiver operating characteristic (ROC) curves for ELISA of SEA, SjPrx-4, SjTPx-1 and combination of SjTPx-1/SjPrx-4 against sera from endemic negative (n = 41) and schistosomiasis-positive by Kato-Katz (n = 30). [file 13071_2020_4313_MOESM4_ESM.tiff]

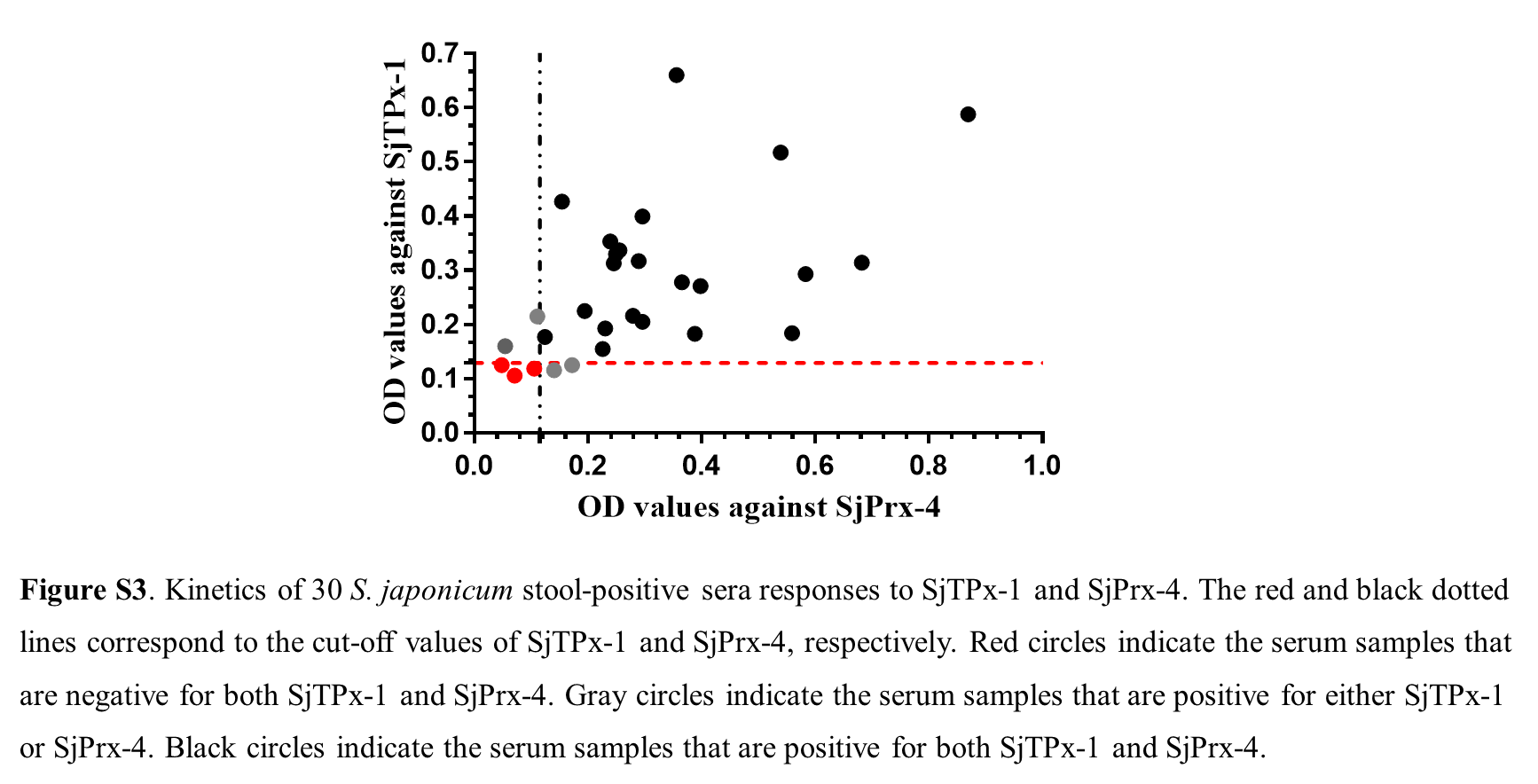

Supplement: Supplementary file 5 — Additional file 5: Figure S3. Kinetics of 30 S. japonicum stool-positive sera response to SjTPx-1 and SjPrx-4. [file 13071_2020_4313_MOESM5_ESM.tiff]
