# Supplementary material for: Analyses of the expression, immunohistochemical properties and serodiagnostic potential of Schistosoma japonicum peroxiredoxin-4
Source: Parasit Vectors. 2020 Sep 1;13:436. doi: 10.1186/s13071-020-04313-w (PMC7460784; doi:10.1186/s13071-020-04313-w)
Supplement: Supplementary file 3 — Additional file 3: Table S2. Statistical analysis of ELISA results of rSjTpx-1, rSjPrx-4 and a combination of rSjPrx-4/rSjTPx-1. [file 13071_2020_4313_MOESM3_ESM.docx]

**Additional file 3: Table S2**. Statistical analysis of ELISA results of SEA, rSjTpx-1, rSjPrx-4 and combination of rSjPrx-4/rSjTPx-1

| Antigen | Sensitivity (%) | Specificity (%) | PPV (%) | NPV (%) | Kappa |
| --- | --- | --- | --- | --- | --- |
| SEA  SjTPx-1 | 90.0  83.3 | 70.7  97.6 | 69.2  96.2 | 90.6  88.9 | 0.58  0.82 |
| SjPrx-4  SjPrx-4/SjTPx-1 | 83.3  90.0 | 87.8  92.7 | 83.3  90.0 | 83.3  92.7 | 0.71  0.83 |

PPV positive predictive values; NPV negative predictive values
